# Effects of Prone Ventilation on Oxygenation, Inflammation, and Lung Infiltrates in COVID-19 Related Acute Respiratory Distress Syndrome: A Retrospective Cohort Study

**DOI:** 10.3390/jcm9124129

**Published:** 2020-12-21

**Authors:** Rohit Khullar, Shrey Shah, Gagandeep Singh, Joseph Bae, Rishabh Gattu, Shubham Jain, Jeremy Green, Thiruvengadam Anandarangam, Marc Cohen, Nikhil Madan, Prateek Prasanna

**Affiliations:** 1 Renaissance School of Medicine and Department of Biomedical Informatics, Stony Brook University, Stony Brook, NY 11794, USA; Joseph.Bae@stonybrookmedicine.edu; 2 Division of Pulmonary Critical Care, Department of Internal Medicine, Newark Beth Israel Medical Center, Newark, NJ 07112, USA; Shrey.Shah@rwjbh.org (S.S.); Thiruvengadam.Anandarangam@rwjbh.org (T.A.); Nikhil.Madan@rwjbh.org (N.M.); 3 Department of Radiology, Newark Beth Israel Medical Center, Newark, NJ 07112, USA; Gagan32092@gmail.com (G.S.); RGattu3@gmail.com (R.G.); Jeremy.Green@rwjbh.org (J.G.); 4 Department of Computer Science, Stony Brook University, Stony Brook, NY 11794, USA; Jain@cs.stonybrook.edu; 5 Division of Cardiology, Department of Internal Medicine, Newark Beth Israel Medical Center, Newark, NJ 07112, USA; Marc.Cohen@rwjbh.org; 6 Department of Biomedical Informatics, Stony Brook University, Stony Brook, NY 11794, USA

**Keywords:** severe acute respiratory syndrome-related coronavirus 2 (SARS-CoV-2), coronavirus disease 2019 (COVID-19), prone ventilation, acute respiratory distress syndrome (ARDS), prone positioning, diagnostic radiology, chest imaging

## Abstract

Patients receiving mechanical ventilation for coronavirus disease 2019 (COVID-19) related, moderate-to-severe acute respiratory distress syndrome (CARDS) have mortality rates between 76–98%. The objective of this retrospective cohort study was to identify differences in prone ventilation effects on oxygenation, pulmonary infiltrates (as observed on chest X-ray (CXR)), and systemic inflammation in CARDS patients by survivorship and to identify baseline characteristics associated with survival after prone ventilation. The study cohort included 23 patients with moderate-to-severe CARDS who received prone ventilation for ≥16 h/day and was segmented by living status: living (*n* = 6) and deceased (*n* = 17). Immediately after prone ventilation, PaO_2_/FiO_2_ improved by 108% (*p* < 0.03) for the living and 150% (*p* < 3 × 10^−4^) for the deceased. However, the 48 h change in lung infiltrate severity in gravity-dependent lung zones was significantly better for the living than for the deceased (*p* < 0.02). In CXRs of the lower lungs before prone ventilation, we observed 5 patients with confluent infiltrates bilaterally, 12 patients with ground-glass opacities (GGOs) bilaterally, and 6 patients with mixed infiltrate patterns; 80% of patients with confluent infiltrates were alive vs. 8% of patients with GGOs. In conclusion, our small study indicates that CXRs may offer clinical utility in selecting patients with moderate-to-severe CARDS who will benefit from prone ventilation. Additionally, our study suggests that lung infiltrate severity may be a better indicator of patient disposition after prone ventilation than PaO_2_/FiO_2_.

## 1. Introduction

Severe acute respiratory syndrome-related coronavirus 2 (SARS-CoV-2) infection causes mild disease in most but may lead to severe disease and acute respiratory distress syndrome (ARDS) [1]. The early phenotype of coronavirus disease 2019 (COVID-19) related ARDS (CARDS) is hypoxemia without overt dyspnea, a heterogeneous pattern of ventilation-perfusion mismatch, shunt physiology, and radiographic findings of bilateral ground-glass opacities and low lung weight from edema formation [2,3]. Some CARDS patients progress to a pattern typical of moderate-to-severe ARDS requiring mechanical ventilation [2,3].

COVID-19 patients requiring mechanical ventilation have high mortality [4], with rates exceeding those of non-COVID-19 ARDS cases [5]. In the New York area, the mortality rate for COVID-19 patients receiving mechanical ventilation was 76.4% for patients ages 18–65 and 97.2% for patients older than 65 [6]. Globally, intensive care unit (ICU) mortality rates have been reported between 26–62% [7,8,9]. Higher mortality of COVID-19 patients may be partially attributed to higher incidences of barotrauma and ventilator-induced lung injury (VILI) [10].

Management guidelines for moderate-to-severe ARDS now recommend early consideration of prone positioning (proning) during mechanical ventilation due to strong evidence of a survival benefit [11,12]. Proning works by reducing ventral-dorsal trans-pulmonary pressure differences [13], reducing lung compression by the heart and diaphragm [14,15,16], and improving lung perfusion [17]. Proning has been demonstrated to improve oxygenation [18], reduce the incidence of VILI [19,20,21], and, in some clinical trials and meta-analyses, lower mortality rates [18,22,23,24,25]. Early studies of prone ventilation in CARDS patients have reported improvement in the ratio of the partial pressure of arterial oxygen (PaO_2_) to the fraction of inspired oxygen (FiO_2_) (PaO_2_/FiO_2_) [26], lung compliance [26], and increased lung recruitability [27].

Here, we compared the effects of prone ventilation on patient oxygenation, lung infiltrates, and systemic inflammation in a cohort of patients with moderate-to-severe CARDS by patient survival. By better understanding the multifactorial effects of prone ventilation in the setting of CARDS, we aimed to identify clinical and radiological factors associated with patient survival after prone ventilation.

## 2. Experimental Section

### 2.1. Study Design and Patient Selection

This retrospective cohort study consists of confirmed SARS-CoV-2-positive adults admitted to the tertiary care center at Newark Beth Israel Medical Center (Newark, NJ, USA) between March and May 2020. SARS-CoV-2 testing was performed by RT-PCR of nasopharyngeal swabs at admission. We abstracted patient data from the electronic medical record (EMR) and grouped patients by their living status (“living” or “deceased”) 28 d post-admission.

Patients were eligible if they were age ≥18, received invasive mechanical ventilation, and met the Berlin definition for moderate-to-severe ARDS: a PaO_2_/FiO_2_ <200 mmHg with positive end-expiratory pressure (PEEP) ≥5 cm H_2_O [11]. Patients must have received ≥16 consecutive hours of prone ventilation for ≥1 d. Patients were excluded if prone ventilation was not tolerated or deemed unsafe. The protocol for prone ventilation is described in Appendix C. This study was approved by the Newark Beth Israel Institutional Review Board (IRB): IRB # 2020.11.

### 2.2. Outcomes

**Figure 1:**
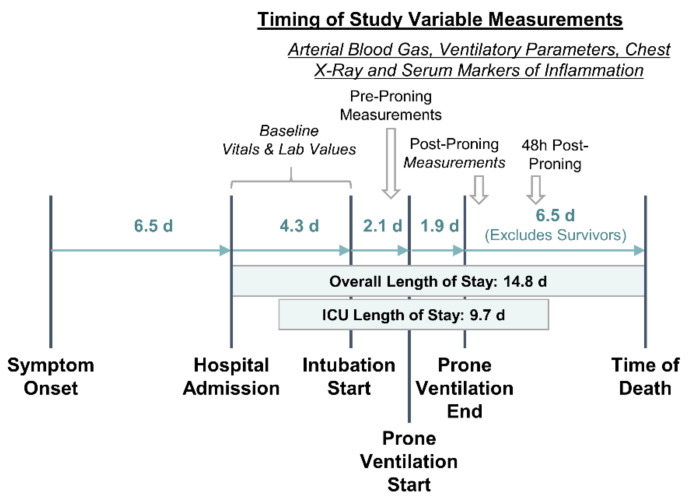
Hospital length of stay, clinical course, and timing of variable measurements. Pre-proning measurements were taken the morning that prone ventilation was initiated. Post-proning measurements were taken within a few hours of cessation of prone ventilation. All time interval durations are presented in days (d) as mean values for the overall study cohort. The interval between prone ventilation end and time of death excluded survivors.

The primary outcome measures in this study were the change in PaO_2_/FiO_2_ and change in lung infiltrate severity score (see “Radiograph Image Analysis” subsection) following prone ventilation. Changes were measured immediately after and 48 h after the final session of prone ventilation relative to the baseline before proning. Figure 1 illustrates the timing of measurements with respect to the clinical course of patients.

Changes in the following arterial blood gas (ABG) values and ventilatory parameters after prone ventilation were also measured: PaO_2_, FiO_2_, PEEP, respiratory rate on mechanical ventilation (RR), and tidal volume (TV) per ideal body weight (IBW) (TV/IBW). RR and TV/IBW were measured immediately after prone ventilation while PaO_2_, FiO_2_, and PEEP were measured both immediately after and 48 h after prone ventilation.

Additional outcome measures included post-prone ventilation changes in Sequential Organ Failure Assessment (SOFA) score, Simplified Acute Physiology Score (SAPS), and the following serological markers of inflammation: C-reactive protein (CRP), D-dimer, ferritin, lactate dehydrogenase (LDH), and procalcitonin.

### 2.3. Data Collection and Definitions

In addition to outcome measures, the following data were abstracted: patient age and demographics (age, sex, ethnicity, BMI), symptoms at admission (dyspnea, fever, cough, weakness, diarrhea), comorbidities (diabetes mellitus, hypertension, chronic kidney disease, lung disease, coronary artery disease, and congestive heart failure), vital signs (blood pressure, heart rate, respiratory rate, temperature, and O_2_ percent saturation), cell counts (white blood cell count and lymphocyte percentage), and critical plasma and serological lab values. Comorbidities were categorized as follows: chronic lung disease, chronic kidney disease (plasma creatinine >1.5 mg/dL for >6 months or previously documented diagnosis), diabetes, hypertension, congestive heart failure, and coronary artery disease.

The “pre-proning” value for all study variables was defined as the last recording before prone ventilation. Measures of clinical course and duration were also recorded. These include the time from symptom onset to admission, total hospital length of stay (LOS), total ICU LOS, time from admission to intubation, time from admission to prone ventilation, and number of consecutive days proned.

### 2.4. Radiograph Image Analysis

We developed a severity score on chest radiographs (CXRs) to determine the COVID-19 pneumonia burden in the lungs. CXRs were interpreted in consensus by three expert readers (≥15, ≥3, and ≥2 years of experience, respectively). Abnormal lung opacification represented the extent of disease. Each lung was divided into three zones—upper, middle, and lower—that were all equal in craniocaudal dimension. A severity score was subsequently assigned to each of these zones for all available time points (before, immediately after, and 48 h post proning). The severity scale consisted of three categories: “0” representing no infiltrates, “1” representing ground-glass infiltrates, and “2” representing confluent infiltrates with or without air bronchograms. A sum of scores from the left and right lungs was calculated for every chest radiograph at each time point, for a maximum possible score of 4 for individual lung zones and 12 overall.

This scoring system resembles an experimental CXR score proposed by Borghesi and Maroldi [28,29]. Borghesi and Maroldi, however, proposed a scoring scale that ranges from 0 to 3 for each lung zone, with 2 assigned for confluent infiltrates with interstitial predominance and 3 assigned for confluent infiltrates with alveolar predominance.

### 2.5. Statistical Analysis

We assessed the effects of prone ventilation on PaO_2_/FiO_2_ by pairwise comparison of measures prior to beginning prone ventilation to measures immediately after and 48 h after the cessation of prone ventilation. We performed a Wilcoxon signed-rank test to identify statistically significant changes. A similar methodology was followed for all secondary outcomes. This pairwise analysis was conducted on the overall study cohort and repeated for the living and deceased subgroups to compare the effects of prone ventilation by living status.

We also evaluated patient characteristics and baseline clinical values that may be risk factors for patient mortality after proning. We segmented the study cohort by living status and compared the living to the deceased by the following measures: patient age and demographics, symptoms at admission, comorbidities, vital signs at admission, cell counts, plasma and serological lab values, baseline ABG and ventilatory parameters, and clinical course (see Table 1 for detailed list). A Mann-Whitney U test was performed on continuous variables and a χ^2^ contingency test was performed on dichotomous categorical variables to determine statistically significant differences.

Finally, we examined the influence of initiating prone ventilation early in the course of mechanical ventilation on the rate of sustained improvement in PaO_2_/FiO_2_. Sustained improvement was defined as a ≥10% increase in PaO_2_/FiO_2_ from baseline to 48 h after prone ventilation. First, we categorized the number of days between intubation and proning initiation into “0–1 days” and “≥2 days”. Then, using a χ^2^ contingency test, we assessed the association between this duration and the rate of sustained improvement in PaO_2_/FiO_2_.

All statistical analysis was completed using IBM SPSS Statistics 27 with an *α*-value of 0.05.

## 3. Results

### 3.1. Cohort Identification and Grouping by Living Status

Between March and May 2020, approximately 850 patients were hospitalized with COVID-19, out of which 300 required ICU admission. Of these patients, 25 received prone ventilation. Two patients did not tolerate proning due to hemodynamic instability and were excluded from the analysis.

Then, 28 days post-admission, 6 (26.1%) of the 23 study participants were alive and 17 (73.9%) were deceased. The primary causes of death were shock (*n* = 7) and multi-organ dysfunction (*n* = 10). On average, the deceased died 7 days after starting prone ventilation.

### 3.2. Patient Characteristics before Intubation and Prone Positioning

Table 1 summarizes the baseline clinical measures and parameters by patient living status. No significant differences were identified between the living and the deceased. Overall, patients were most likely to be male (65.2%), self-identified as African American or Hispanic (91.3%), obese (median BMI, 31; range, 22–45), and aged 57 years (median; range, 25–75). Before proning, most patients were categorized with severe ARDS with a median PaO_2_/FiO_2_ of 76 mmHg (range, 35–190 mmHg). None of the living were hypoxemic (PaO_2_ < 60 mmHg) under mechanical ventilation while 5 (29.4%) of the deceased were hypoxemic.

### 3.3. Patient Response to Prone Ventilation

**Figure 2:**
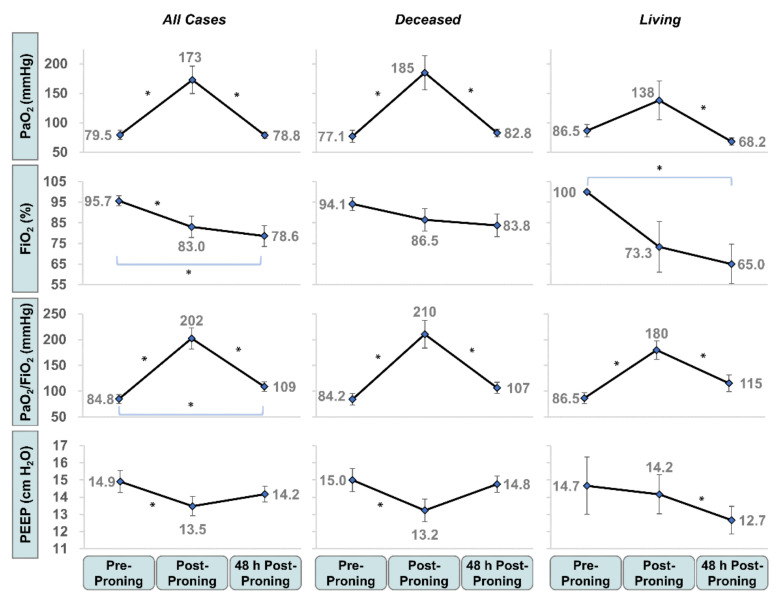
Change in arterial blood gas (ABG) and ventilatory parameters before, after, and 48 h after prone ventilation by living status. Mean PaO_2_, FiO_2_, PaO_2_/FiO_2_ and positive end-expiratory pressure (PEEP) are presented before proning, immediately post-proning, and 48 h post-proning. Patients segmented by living status 28 d post-admission. Error bars represent S.E. of the mean. * Denotes statistical significance, given by the Wilcoxon signed-rank test with α = 0.05.

Prone ventilation was generally well tolerated with 48% of the cohort proned for ≥2 d and 26% proned for ≥3 d. We demonstrated marked improvement in oxygenation and ventilatory parameters at the end of prone ventilation. PaO_2_ and PaO_2_/FiO_2_ increased by 117% (*p* < 3 × 10^−4^) and 139% (*p* < 3 × 10^−5^), respectively. FiO_2_ and PEEP decreased by 13% (*p* < 0.05) and 10% (*p* < 0.02), respectively (Figure 2). As seen in Table 2, we also observed a significant reduction in mean respiratory rate (27.2 to 23.6 breaths per minute; *p* < 0.007).

At 48 h after prone ventilation, FiO_2_ (*p* < 0.02) and PaO_2_/FiO_2_ (*p* < 0.05) remained significantly improved over baseline (Figure 2). The mean FiO_2_, after initially reducing to 83.0%, continued to improve to 78.6% after 48 h. The mean PaO_2_/FiO_2_, after initially increasing, reverted towards but remained significantly higher than baseline at 109 mmHg (84.8 to 109 mmHg; *p* < 2 × 10^−4^).

Both the living and the deceased showed improvement in PaO_2_/FiO_2_ immediately after prone ventilation (Figure 2). The increase was greater for the deceased (84.2 to 210 mmHg; *p* < 3 × 10^−4^) than for the living (86.5 to 180 mmHg; *p* < 0.03). After 48 h, neither the living nor the deceased maintained a significant improvement in PaO_2_/FiO_2_. After prone ventilation, the living were administered mechanical ventilation with significantly lower PEEP (12.7 vs. 14.8 cmH_2_O, *p* < 0.05) and FiO_2_ (78.6 vs. 83.8%, *p* < 0.06) than the deceased.

### 3.4. Patients with Sustained Improvement in PaO_2_/FiO_2_

**Figure 3:**
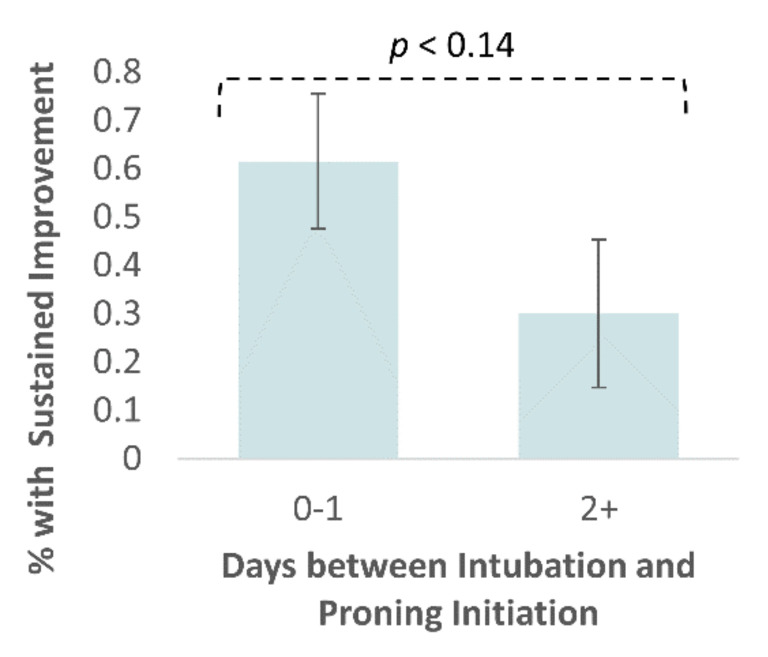
Sustained improvement in PaO_2_/FiO_2_ by days between intubation and proning initiation. Sustained improvement was defined as a ≥10% improvement in PaO_2_/FiO_2_ between pre-proning measurement and measurement 48 h after proning initiation. Patients were segmented by the timing of proning initiation relative to the timing of intubation. Error bars represent the S.E. of the mean. Statistical significance is given by the χ-squared contingency test with α = 0.05.

At 48 h after prone ventilation, 11 (47.8%) patients demonstrated sustained improvement in PaO_2_/FiO_2_. Those proned earlier (<2 d) into their course of mechanical ventilation were twice as likely to show sustained improvement in PaO_2_/FiO_2_ after 48 h (*p* < 0.14) (Figure 3). A total of 61.5% of patients who were proned <2 d after intubation maintained PaO_2_/FiO_2_ ≥ 10% of their baseline value after 48 h versus 30.0% for patients who were proned ≥2 d after intubation.

### 3.5. Evaluation of Lung Infiltrates on Chest Radiographs

**Figure 4:**
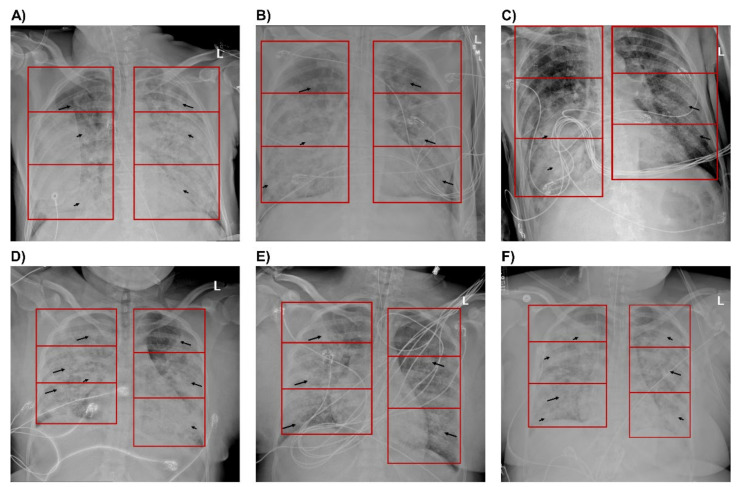
Chest X-rays of patients with sustained improvement in PaO_2_/FiO_2_ and with decline in PaO_2_/FiO_2_ after 48 hours (h). Anterior-Posterior (AP) radiographs of the chest with lung zones demarcated by a red box. Images (**A**–**C**) are from a single patient with sustained improvement in PaO_2_/FiO_2_. Images (**D**–**F**) are from a single patient with a decline in PaO_2_/FiO_2_. (**A**) Image captured 48 h before proning demonstrates ground-glass opacities in the left and right upper lung zones (long arrows), with confluent consolidations in the right middle, right lower, left middle, and left lower zones (short arrows). Images (**B**) immediately after proning demonstrate improving infiltrates bilaterally with significant improvement in the left middle, left lower, and right lower lung zones, and (**C**) 48 h after proning demonstrate significant improvement in the infiltrates bilaterally with residual dense consolidation in the right middle and lower lung zones. (**D**) Image one day before proning demonstrates ground-glass opacities throughout all lung zones (long arrows), with diffusely scattered confluent consolidations bilaterally (short arrows). Images (**E**) immediately after proning demonstrate improving infiltrates, particularly in the right upper, left upper, and left middle lung zones, and (**F**) 48 h after proning demonstrate worsening of infiltrates bilaterally with increased confluent consolidations in the left and right upper lung zones.

Figure 4 illustrates a series of chest radiographs for two study participants captured before, immediately after, and 48 h after prone ventilation. Figure 4A–C are representative of the progression of patients with sustained improvement in PaO_2_/FiO_2_: confluent consolidations in lower and middle lung zones and ground-glass opacities (GGOs) in upper lung zones before prone ventilation and bilateral improvements in most lung zones 48 h after prone ventilation. Figure 4D–F are representative of the progression for patients who did not show sustained improvement in PaO_2_/FiO_2_: GGOs with diffusely scattered confluent consolidations throughout all lung zones before prone ventilation, temporal improvement immediately after prone ventilation, and bilateral worsening of infiltrates 48 h after prone ventilation.

We applied our CXR scoring system to quantify the severity of lung infiltrates and observed an inverse relationship between lung height and lung severity scores. Lung severity scores in the lower lung zones of CXRs captured before prone ventilation were most indicative of patient disposition after prone ventilation. Four of 5 patients with bilateral confluent infiltrates in lower lung zones (lung severity score = 4.0) were alive after prone ventilation while only 1 of 12 patients with bilateral GGOs (lung severity score = 2.0) were alive. Conversely, only 1 of 5 patients with bilateral confluent infiltrates in lower lung zones showed a sustained improvement in PaO_2_/FiO_2_ after 48 h while 7 of 12 patients with bilateral GGOs showed sustained improvement.

**Figure 5:**
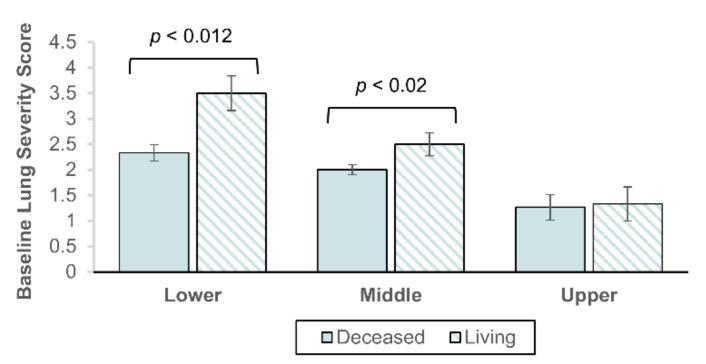
Mean lung severity score before prone ventilation by patient living status 28 days post-admission. Lung severity score measured by 3 experienced radiologists in chest X-rays captured the day before prone ventilation. Shown by lower, middle, and upper lung zones, summed for the left and right lung. Error bars represent S.E. of the mean. Statistical significance is given by the Mann-Whitney U test with α = 0.05.

Next, we compared lung severity scores in CXRs captured before prone ventilation was initiated between the living and deceased. As seen in Figure 5, the living were observed with significantly higher lung severity scores than the deceased in the lower (3.5 vs. 2.3; *p* < 0.012) and middle (2.5 vs. 2.0; *p* < 0.02) lung zones. No significant difference was observed in the upper lung zones (1.3 vs. 1.3; *p* < 0.73).

**Figure 6:**
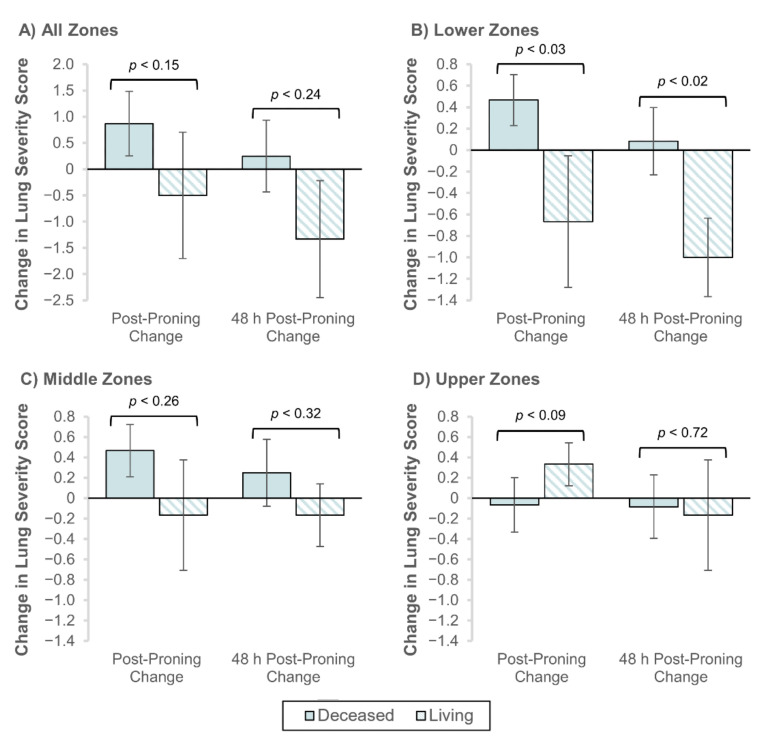
Change in lung severity scores immediately and 48 hours (h) post-proning by living status. The change from baseline in lung severity score was measured in radiographs taken immediately and 48 h after prone ventilation. Results are shown by the following aggregate lung zones: (**A**) all 6 lung zones, (**B**) lower lung zones, (**C**) middle zones, and (**D**) upper zones. Error bars represent S.E. of the mean. Statistical significance is given by the Mann-Whitney U test with α = 0.05.

Lastly, we compared the change in lung severity scores after prone ventilation for the living and deceased. Figure 6A illustrates that both immediately after and 48 h after prone ventilation, the overall lung severity score worsened for the deceased and improved for the living. This pattern of change was consistent for the lower and middle lung zones (Figure 6B,C), but not for the upper lung zones (Figure 6D).

At the patient level, we did not observe statistically significant changes in lung severity scores after prone ventilation (Table A1). However, lung severity scores in the lower lung zones improved significantly for the living relative to the deceased, both immediately after (−0.67 vs. 0.47; *p* < 0.03) and 48 h after (−1.0 vs. 0.08; *p* < 0.02) prone ventilation (Figure 6B).

### 3.6. Pre-Proning Characteristics of Patients with Sustained Improvement in PaO_2_/FiO_2_

At 48 h after prone ventilation, 11 (47.8%) study participants demonstrated sustained improvement in PaO_2_/FiO_2_. These patients had significantly lower mean serum values for creatinine AST (0.79 vs. 1.32 IU/L; *p* < 0.04), AST (55 vs. 84 IU/L; *p* < 0.04), and ferritin (992 vs. 1863 μg/L; *p* < 0.01) than those who did not show sustained improvement in PaO_2_/FiO_2_ (Table A2).

### 3.7. Proning Effects on Serological Markers of Inflammation

In addition to ABG and ventilatory markers, significant reductions in inflammatory serological values and significant improvements in SOFA and SAPS scores were observed in patients following prone ventilation (Table 2). After prone ventilation, mean LDH decreased from 986 to 840 U/L (*p* < 0.04), mean CRP decreased from 13.8 to 8.5 mg/L (*p* < 0.05), and mean ferritin decreased from 1672 to 1195 μg/L (*p* < 0.03). Mean SOFA scores improved from 4.65 to 3.78 (*p* < 5 × 10^−4^) and mean SAPS scores improved from 32.6 to 29.9 (*p* < 5 × 10^−6^). No significant differences in mean procalcitonin nor D-dimer were observed following prone ventilation.

## 4. Discussion

This study was conducted at the peak of the first wave of the COVID-19 pandemic near an early epicenter in New York City. An overwhelming number of patients with SARS-CoV-2 infection was encountered in April at Newark Beth Israel Medical Center. Approximately 35% required ICU admission for moderate-to-severe ARDS, 2–3 times the rate reported by other institutions [6,7,30]. The mortality of intubated patients seemed high relative to usual ARDS patients despite treatment with lung-protective ventilation. Patients who matched the criteria for prone ventilation underwent the procedure as per hospital protocol. Their response to proning was observed in this study. The strengths of this study include the assessment of proning effects up to 48 h and our consideration of effects on multiple parameters for a single cohort, including changes in arterial oxygenation, ventilatory parameters, serological markers of inflammation, and lung infiltrates as observed by CXR.

The favorable response to prone ventilation that we observed among the study population corresponds to previously published studies of prone ventilation of CARDS patients [26,27,31] and ARDS patients in the PROSEVA trial [18], including significant improvements in PaO_2_, PaO_2_/FiO_2_, FiO_2_, PEEP, and RR immediately after proning and sustained improvements in FiO_2_ (*p* < 0.02) and PaO_2_/FiO_2_ (*p* < 0.05) 48 h after proning,. Despite these improvements, most patients remained in a moderate-to-severe ARDS state after 48 h and 73.9% were deceased within 28 days post-admission.

Improvement in PaO_2_/FiO_2_ was not associated with increased survival after prone ventilation. In fact, PaO_2_/FiO_2_ improved to a greater degree amongst the deceased than the living. However, using the density of radiographic opacities in CXRs as a correlate for disease burden, we observed simultaneous improvement of pulmonary infiltrates among the living and worsening of pulmonary infiltrates among the deceased (*p* < 0.02). These changes were most prominent in the lower lung zones and in regions of dense consolidation. A recently published study of 9 early CARDS patients proned within 3 days following intubation also reported significant improvement in PaO_2_/FiO_2_ after proning [31]. The authors noted a significant reduction in lung opacity in CXRs captured between 3 to 16 h after intubation but did not measure a significant difference in lung opacity after proning. This may be related to their focus on the overall lung rather than on gravity-dependent lung zones that display increased density on CT imaging in ARDS patients and density redistribution after proning [31,32,33].

Prior studies have associated increase in oxygenation after prone ventilation to the recruitment of atelectatic airspaces, increased ventilation of gravity-dependent segments, and equalized aeration along the dorsal-ventral axis [19,33,34,35]. However, they too indicate that improved arterial oxygenation does not correlate with survival [20,36]. The current hypothesis is that the survival benefit of proning is attributed to protection against lung injury [19,37]. Understanding the effects of changes in clinical parameters on imaging progression may improve feature interpretability in prediction modeling on CXR/CT, which so far has largely depended purely on the imaging aspects [38,39,40,41] and often ignored associated inflammatory and oxygenation parameters.

Radiological patterns of lower lung infiltrate severity in CXRs captured before prone ventilation may correlate with patient survival after prone ventilation. 80% of patients with bilateral dense infiltrates survived versus 8% for patients with bilateral GGOs. The reverse trend was seen for oxygenation: 20% of patients with dense infiltrates and 42% of patients with GGOs showed a sustained improvement in PaO_2_/FiO_2_. A recent retrospective case-control study of 51 patients with ARDS following surgery for intraabdominal infection yielded similar results. Prone ventilation was associated with significantly higher survival in patients with dorsal lung atelectasis (equivalent to dense infiltrates by our methodology) while no such survival benefit was seen in patients with GGOs [42]. This finding is consistent with studies that have demonstrated a more pronounced response to prone ventilation in patients with lobar infiltrates [43].

While further investigation is needed to establish its ability to predict patient disposition after prone ventilation, we offer a method for quantitatively assessing lung infiltrate severity in CXRs captured near the time of intubation that may facilitate early proning in the management of CARDS patients. Our method resembles scoring systems reported in prior literature [28,29] but simplifies the procedure for scoring CXRs displaying confluent infiltrates; as a result of this adaptation, radiologists would not be required to distinguish between confluent infiltrates of interstitial or alveolar predominance. We believe this may lead to a more consistent interpretation of CXR findings, thereby increasing reproducibility and improving implementation of the score in a larger, multi-center study.

Early proning may be an important consideration for future management of CARDS patients. We observed that patients proned <2 d after initiating mechanical ventilation were twice as likely to show a sustained response in PaO_2_/FiO_2_ than those proned ≥2 d after initiating mechanical ventilation. Although not determined to be statistically significant (*p* < 0.14)—potentially due to the study size—this result is consistent with recent recommendations that CARDS patients be proned as early as the pre-intubation phase [2,44]. The time between intubation and proning has not been a focus of prior studies, but early proning (within 48 h following endotracheal intubation) was a feature of the PROSEVA protocol [18]. Several small, uncontrolled, observational studies have been published recently evaluating proning before intubation, but so far have not provided conclusive evidence supporting the practice in CARDS patients [45,46,47,48,49]. Clinical trials are in progress and may assist in providing stronger support for proning before intubation (NCT04325906, NCT04383613, NCT04359797, NCT03095300, NCT04350723, and NCT04347941).

The overall mortality in this study was analogous to a study conducted at a New York hospital group over a similar timeframe [6] but exceeded historical mortality rates for severe ARDS [5]. Higher mortality in CARDS may be attributed to overwhelmed healthcare systems and lack of specific virus-directed treatment. Historically, 10% of ICU admissions are diagnosed with ARDS; a reported 19% of COVID-19 patients and 47–88% of COVID-19 ICU admissions required invasive mechanical ventilation due to respiratory failure [5,7,30]. Additionally, we observed slightly higher TV/IBW than recommended for ARDS patients (7 mL/kg median vs. 6 mL/kg recommended), which may have increased the likelihood for VILI.

Lastly, we speculated whether prone positioning may play a role in reducing systemic inflammation, in part by enhancing alveolar fluid clearance [19,50]. Inflammatory responses during the pathogenesis of ARDS or secondary to VILI may be associated with pulmonary and extra-pulmonary organ dysfunction and strategies to reduce inflammation may result in increased survival [36]. In a prior study, prone ventilation was associated with reduced IL-6 concentrations in bronchoalveolar lavage fluid and plasma; reduced plasma levels of IL-6 were associated with improved survival in ARDS patients [51]. Another study of ARDS patients receiving conventional lung-protective mechanical ventilation reported reduced cytokine levels and neutrophil counts after proning and increased cytokine levels when patients were transferred from conventional lung-protective ventilation to high-frequency oscillatory ventilation in either the prone or supine position [52]. We examined serological markers of inflammation commonly evaluated during critical care management, including LDH, procalcitonin, CRP, D-dimer, and ferritin. Elevation of LDH is a suggested risk factor for critical illness following SARS-CoV-2 infection [53]. Following prone ventilation, we observed significant reductions in LDH, CRP, and ferritin but did not see an associated survival benefit.

The primary limitation of this study was our inability to identify a control group for CARDS patients who received mechanical ventilation without prone positioning over a similar timeframe. All patients who met the proning criteria set by the institution received prone ventilation. Those who did not receive prone ventilation tended to be sicker or more hemodynamically unstable and would not be suitable for comparison. This prevented us from assessing the mortality benefit of prone ventilation in CARDS patients. Additionally, most patients tolerated only one day of prone ventilation. This contrasts with recent studies that reported prone ventilation of ≥16 h per day for between 4–10 consecutive days [19].

Other limitations include the small sample size, involvement of a single medical center, and relatively homogeneous sample of patients, as well as the proportional inequity of survivors to the deceased. The patient population displayed relatively distinct and consistent age, demographic, and baseline characteristics with a high frequency of risk factors for severe disease and respiratory failure from SARS-CoV-2 infection [53]. These factors may limit the generalizability of our findings. Further investigation with a larger cohort and multivariable analysis of potentially relevant factors from the patient history, clinical and laboratory values, and radiographic features is needed to better guide clinicians on the use of early proning in CARDS patients.

## 5. Conclusions

This study offers a first look at the simultaneous effects of prone ventilation on patient oxygenation and ventilation, lung infiltrates observed in chest radiographs, and systemic inflammation for a cohort of CARDS patients with moderate-to-severe ARDS severity. We propose a method of quantifying the disease burden of CARDS from the density of opacities in CXRs. Our analysis of lung infiltrate severity in chest radiographs captured before prone ventilation provides initial evidence of the clinical utility of CXRs in predicting the disposition of CARDS patients after prone ventilation. Future studies will involve incorporating CXR-derived lung severity scores, with other clinical and inflammatory markers, into a machine learning algorithm to select patients who will respond favorably to prone ventilation. Prone positioning is a resource-intensive strategy; during periods of high ICU admission rates, patient stratification with the assistance of a predictive model can help reduce strain on the healthcare team and improve overall patient outcomes. Lastly, our preliminary analysis suggests that changes in pulmonary infiltrates, rather than arterial oxygenation, may be more appropriate in assessing disease progression and clinical success of patients receiving prone ventilation.

## Figures and Tables

**Table 1:** Clinical characteristics, lung infiltrate severity, and pronation timeline by living status.

Characteristic	Deceased (*n* = 17)	Living (*n* = 6)	Total (*n* = 23)
**Demographics**			
Age, Median (Range)	57 (25, 75)	56 (40, 63)	57 (25, 75)
Sex, Female (%)	6 (35.3)	2 (33.3)	8 (34.8)
BMI, Median (Range), kg/m^2^	30 (23, 42)	36 (22, 45)	31 (22, 45)
Race/Ethnicity, Count (% Distribution)			
African American	9 (52.9)	3 (50.0)	12 (52.2)
Hispanic	7 (41.2)	2 (33.3)	9 (39.1)
American Indian	-	1 (16.7)	1 (4.3)
Asian	1 (5.9)	-	1 (4.3)
**Symptoms and Comorbidities at Admission**			
Symptoms (%)			
Dyspnea	15 (88.2)	6 (100.0)	21 (91.3)
Fever	14 (82.4)	4 (66.7)	18 (78.3)
Cough	12 (70.6)	3 (50.0)	15 (65.2)
Weakness	16 (94.1)	6 (100.0)	22 (95.7)
Diarrhea	1 (5.9)	1 (16.7)	2 (8.7)
^†^ Comorbidities (%)			
Diabetes Mellitus	7 (41.2%)	2 (33.3%)	9 (39.1%)
Hypertension	8 (47.1%)	3 (50.0%)	11 (47.8%)
Congestive Heart Failure	1 (5.9%)	0 (0%)	1 (4.3%)
**Clinical Values before Prone Ventilation, Median (Range)**			
Vitals			
Blood Pressure—Systolic (mmHg)	125 (109, 153)	119.5 (110, 167)	124 (109, 167)
Blood Pressure—Diastolic (mmHg)	75 (49, 93)	62.5 (52, 87)	72 (49, 93)
Heart Rate (Beats/min)	105 (66, 125)	96.5 (53, 118)	102 (53, 125)
Respiratory Rate (Breaths/min)	28 (20, 34)	25 (21, 35)	26 (20, 35)
Temperature (°F)	99.7 (97, 102.6)	99.5 (98, 100.3)	99.7 (97, 102.6)
O_2_% Saturation	92 (87, 100)	92 (88, 98)	92 (87, 100)
Lab Values			
Blood Urea Nitrogen (mg/dL)	28 (13, 104)	26 (14, 32)	28 (13, 104)
Creatinine (IU/L)	0.91 (0.54, 4.68)	1.47 (0.44, 2.2)	0.97 (0.44, 4.68)
Lactate (mg/dL)	1.8 (0.7, 6.7)	1.8 (1.2, 4.9)	1.8 (0.7, 6.7)
Troponin (ng/mL)	0.12 (0.015, 0.71)	0.055 (0.015, 0.28)	0.081 (0.015, 0.71)
LDH (U/L)	958 (535, 1875)	888.5 (621, 1757)	958 (535, 1875)
Procalcitonin (ng/mL)	0.92 (0.16, 200)	0.78 (0.41, 24.81)	0.92 (0.16, 200)
C-Reactive Protein (mg/L)	9.52 (1.46, 34)	14.6 (0.34, 24.1)	11.1 (0.34, 34)
D-Dimer (ng/mL)	13.8 (0.96, 35.78)	23.2 (1.8, 35.78)	13.8 (0.96, 35.78)
Fibrinogen (mg/dL)	502 (100, 679)	290 (165, 572)	483 (100, 679)
Ferritin (μg/L)	1151 (298, 5509)	1308 (992, 2198)	1285 (298, 5509)
Sedimentation Rate (mm/h)	40 (5, 107)	26 (7, 72)	39 (5, 107)
White Blood Cell Count (1000/mm^3^)	13.6 (6.1, 31.7)	11.2 (7.1, 26.8)	13.3 (6.1, 31.7)
Lymphocyte Percent	0.05 (0.02, 0.15)	0.04 (0.03, 0.08)	0.05 (0.02, 0.15)
**ABG and Ventilatory Parameters, Supine, Median (Range)**			
PaO_2_ (mmHg)	66.0 (35, 190)	77.5 (66, 138)	73.0 (35, 190)
FiO_2_ (%)	100 (50, 100)	100 (100, 100)	100 (50, 100)
PaO_2_/FiO_2_ (mmHg)	76.0 (35, 190)	77.5 (66, 138)	76.0 (35, 190)
PEEP (cm H_2_O)	15.0 (10, 20)	15.0 (10, 20)	15.0 (10, 20)
Respiratory Rate	24 (16, 30)	24 (20, 26)	24 (16, 30)
Tidal Volume/IBW (mL/kg)	7.0 (5.8, 8.3)	7.4 (5.3, 8.0)	7.0 (5.3, 8.3)
**Pronation Timeline and Length of Stay (In Days)**			
Time to Intubation, Median (Range)			
From Admission	3.0 (0, 15)	3.5 (0, 5)	3.0 (0, 15)
From Symptom Onset	7.0 (2, 14)	7.0 (5, 14)	7.0 (2, 14)
Time to Pronation, Median (Range)			
From Admission	5.0 (2, 18)	4.5 (2, 17)	5.0 (2, 18)
From 1st Symptom Appearance	11.0 (7, 25)	13.0 (9, 24)	12.0 (7, 25)
From Intubation	1.0 (0, 7)	2.0 (0, 13)	1.0 (0, 13)
Days Proned	1 (1, 4)	1.5 (1, 6)	1 (1, 6)
Time to Death After Pronation	7 (2, 16)	-	7 (2, 16)

No statistically significant differences observed (given by Mann-Whitney U test with α = 0.05); ^†^ No patients presented with chronic kidney disease, chronic lung disease, or coronary artery disease.

**Table 2:** Change in clinical measures and outcomes immediately post-proning.

Characteristic	Pre-Proning	Post-Proning	Δ * (Post−Pre)	*p*-Value ^†^
**Arterial Blood Gas and Ventilatory Markers, Mean Values**				
^‡^ Respiratory Rate (bpm)	27.2	23.6	(3.6)	0.006
^‡, §^ Tidal Volume/IBW (mL/kg)	7.1	7.0	(0.1)	0.109
**Patient Scores, Mean Values**				
SOFA	4.78	3.65	(1.13)	4 × 10^−4^
SAPS	32.57	29.91	(2.65)	4 × 10^−5^
**Inflammatory Markers, Mean Values**				
LDH	986	840	(146)	0.03
Procalcitonin	12.5	1.61	(2.29)	0.36
C-Reactive Protein	13.8	8.51	(5.46)	0.04
D-Dimer	19.5	22.1	2.65	0.71
Ferritin	1672	1195	(490)	0.02

* Delta values are calculated at patient level before mean calculation; patients are excluded from mean calculation if missing data at pre or post-proning time point; ^†^ Statistical significance measured using Wilcoxon Signed Rank Test; ^‡^ Measured while under mechanical ventilation; ^§^ IBW = ideal body weight.

## Appendix A

**Table A1:** Mean lung severity scores by lung segment.

Lung Segment	Pre-Proning	Post-Proning	48 h Post-Proning	*p*-Value (Post–Pre)	*p*-Value (48 h Post–Pre)
**All Cases**					
All Lung Segments	6.1	6.5	6.0	0.49	0.77
Lower Lung Zones	2.7	2.8	2.4	0.52	0.39
Middle Lung Zones	2.1	2.4	2.3	0.22	0.62
Upper Lung Zones	1.3	1.3	1.4	0.91	0.66
**Living**					
All Lung Segments	7.3	6.8	6.0	0.50	0.28
Lower Lung Zones	3.5	2.8	2.5	0.33	0.06
Middle Lung Zones	2.5	2.3	2.3	0.79	0.56
Upper Lung Zones	1.3	1.7	1.2	0.16	0.79
**Deceased**					
All Lung Segments	5.6	6.0	6.4	0.16	0.61
Lower Lung Zones	2.3	2.8	2.4	0.07	0.71
Middle Lung Zones	2.0	2.4	2.2	0.08	0.45
Upper Lung Zones	1.3	1.2	1.4	0.71	0.71

Lung severity scores were averaged at the patient level. Mann-Whitney U-Test was performed to measure significance with α = 0.05.

## Appendix B

**Table A2:** Clinical characteristics, pronation timeline, and length of stay by 48 h improvement in PaO_2_/FiO_2_.

Characteristic	Same or Decline (*n* = 12)	48 h Improvement (*n* = 11)	Total (*n* = 23)
**Demographics**			
Age, Median (Range)	58.5 (31, 65)	55 (25, 75)	57 (25, 75)
Sex, Female (%)	3 (25)	5 (45)	8 (34.8)
BMI, Median (Range), kg/m^2^	30.9 (22.4, 45.0)	30.8 (22.5, 39.8)	30.8 (22.4, 45.0)
Race/Ethnicity, Count (% Distribution)	-	-	-
African American	7 (58.3)	5 (45.5)	12 (52.2)
Hispanic	4 (33.3)	5 (45.5)	9 (39.1)
American Indian	0	1 (9.1)	1 (4.3)
Asian	1 (8.3)	0	1 (4.3)
**Symptoms and Comorbidities at Admission**			
Symptoms (%)	-	-	-
Dyspnea	10 (83.3)	11 (100)	21 (91.3)
Fever	10 (83.3)	8 (72.7)	18 (78.3)
Cough	9 (75.0)	6 (54.5)	15 (65.2)
Weakness	12 (100)	10 (90.9)	22 (95.7)
Diarrhea	2 (16.7)	0	2 (8.7)
^†^ Comorbidities (%)	-	-	-
Diabetes Mellitus	4 (33.3)	5 (45.5)	9 (39.1%)
Hypertension	4 (33.3)	7 (63.6)	11 (47.8%)
Congestive Heart Failure	0	1 (9.1)	1 (4.3%)
**Clinical Values before Intubation, Median (Range)**			
Vitals	-	-	-
Blood Pressure—Systolic (mmHg)	125 (109, 167)	124 (110, 153)	124 (109, 167)
Blood Pressure—Diastolic (mmHg)	75.5 (57, 89)	68 (49, 93)	72 (49, 93)
Heart Rate (Beats/min)	97 (66, 121)	105 (53, 125)	102 (53, 125)
Respiratory Rate (Breaths/min)	26 (20, 35)	26 (21, 34)	26 (20, 35)
Temperature (°F)	99.8 (97, 102.6)	99.2 (97.8, 101.4)	99.7 (97, 102.6)
O_2_% Saturation	93 (87, 98)	92 (87, 100)	92 (87, 100)
Lab Values	-	-	-
Glucose (mg/dL)	136 (103, 354)	136 (101, 196)	136 (101, 354)
Sodium (mEQ/L)	142 (135, 158)	143 (138, 151)	142 (135, 158)
Blood Urea Nitrogen (mg/dL)	30.5 (13, 104)	20 (13, 60)	28 (13, 104)
* Creatinine (IU/L)	1.32 (0.72, 4.68)	0.79 (0.44, 2.7)	0.97 (0.44, 4.68)
Lactate (mg/dL)	1.9 (0.7, 6.7)	1.8 (0.7, 2.6)	1.8 (0.7, 6.7)
Troponin (ng/mL)	0.091 (0.015, 0.71)	0.081 (0.015, 0.289)	0.081 (0.015, 0.71)
LDH (U/L)	991 (538, 1757)	733 (535, 1875)	958 (535, 1875)
* AST (IU/L)	84 (54, 342)	55 (24, 144)	73 (24, 342)
ALT (IU/L)	78 (25, 163)	44 (12, 111)	72 (12, 163)
Alkaline Phosphatase (IU/L)	106 (51, 201)	125 (77, 294)	117 (51, 294)
Procalcitonin (ng/mL)	2.51 (0.16, 200)	0.71 (0.41, 2.6)	0.92 (0.16, 200)
C-Reactive Protein (mg/L)	19.4 (1.25, 32.6)	8.65 (0.34, 34)	11.1 (0.34, 34)
D-Dimer (ng/mL)	10.85 (0.96, 35.78)	24.08 (1.8, 35.78)	13.82 (0.96, 35.78)
Fibrinogen (mg/dL)	513 (165, 679)	269 (100, 653)	483 (100, 679)
* Ferritin (μg/L)	1863 (572, 5509)	992 (298, 2198)	1285 (298, 5509)
Total Bilirubin (mg/dL)	0.5 (0.2, 1.8)	0.7 (0.3, 0.9)	0.5 (0.2, 1.8)
Sedimentation Rate (mm/h)	42.5 (7, 107)	17 (5, 100)	39 (5, 107)
White Blood Cell Count (1000/mm^3^)	11.25 (6.1, 21)	17.1 (7, 31.7)	13.3 (6.1, 31.7)
Lymphocyte Percent	5 (2, 11)	4 (3, 15)	5 (2, 15)
**ABG and Ventilatory Parameters, Supine, Median (Range)**			
PAO_2_ (mmHg)	79 (48, 190)	66 (35, 152)	73 (35, 190)
FiO_2_ (%)	100 (50, 100)	100 (80, 100)	100 (50, 100)
P/F Ratio (mmHg)	79 (48, 190)	66 (35, 152)	76 (35, 190)
PEEP (cm H_2_O)	15 (10, 20)	15 (10, 20)	15 (10, 20)
Respiratory Rate	23 (16, 30)	24 (20, 26)	24 (16, 30)
Tidal Volume/IBW (mL/kg)	450 (380, 500)	450 (380, 500)	450 (380, 500)
**Pronation Timeline and Length of Stay (In Days)**			
Time to Intubation, Median (Range)	-	-	-
* From Admission	3 (0, 5)	5 (3, 15)	3 (0, 15)
From Symptom Onset	7 (3, 14)	7 (2, 14)	7 (2, 14)
Time to Pronation, Median (Range)	-	-	-
* From Admission	4 (2, 10)	8 (3, 18)	5 (2, 18)
From 1st Symptom Appearance	11 (7, 17)	13 (8, 25)	12 (7, 25)
From Intubation	2 (0, 7)	1 (0, 13)	1 (0, 13)
Days Proned	1 (1, 6)	2 (1, 3)	1 (1, 6)
Time to Death After Pronation	3 (25.0)	3 (27.3)	3 (26.1)

* Denotes statistical significance, given by Mann-Whitney U test with α = 0.05; ^†^ No patients presented with chronic kidney disease, chronic lung disease, or coronary artery disease.

## Appendix C. Prone Ventilation Protocol

Patients who failed to improve with either high flow or non-invasive ventilation were intubated and treated with tidal volumes of 4–6 mL/kg and conservative fluid management strategy. The ventilator was adjusted to aim for PaO_2_ > 55 mmHg and pH > 7.2. PEEP was titrated using driving pressures to avoid barotrauma. If patients failed to improve despite ventilator optimization, prone ventilation was considered. Patients with hemodynamic instability and morbid obesity (BMI > 35) were excluded.

Patients who met the criteria were proned manually by a team of physicians, respiratory therapists, and nurses. Prone ventilation was continued for 16h before patients were returned to the supine position. Proning was repeated daily based on clinical response and the discretion of the attending physician. Reasons for discontinuation included unstable hemodynamics, worsening renal failure requiring continuous renal replacement therapy, and lack of improvement or worsening in PaO_2_/FiO_2_.

One hundred percent of the study population were treated with hydroxychloroquine and steroids, 96% were treated with tocilizumab, and 83% were administered a neuromuscular blockade agent.
